# Transcriptomic analysis provides insight into the mechanism of IKKβ-mediated suppression of HPV18E6-induced cellular abnormalities

**DOI:** 10.1093/g3journal/jkad020

**Published:** 2023-02-01

**Authors:** Quincy P Collins, Michael J Grunsted, Dahiana Arcila, Yi Xiong, Mojgan Padash Barmchi

**Affiliations:** Department of Biology, University of Oklahoma, Norman, OK 73019, USA; Department of Biology, University of Oklahoma, Norman, OK 73019, USA; Department of Biology, University of Oklahoma, Norman, OK 73019, USA; Department of Ichthyology, Sam Noble Oklahoma Museum of Natural History, Norman, OK 73019, USA; Department of Microbiology and Plant Biology, University of Oklahoma, Norman, OK 73019, USA; Department of Biology, University of Oklahoma, Norman, OK 73019, USA

**Keywords:** *Drosophila*, HPV, E6, UBE3A, transcriptome, IKKβ, cancer, differential gene expression

## Abstract

High-risk human papillomaviruses (HPVs) 16 and 18 are responsible for more than 70% of cervical cancers and majority of other HPV-associated cancers world-wide. Current treatments for these cancers have limited efficacy, which in turn has resulted in disease recurrence and poor survival rates in advanced disease stages. Hence, there is a significant need for development of novel molecularly-targeted therapeutics. This can only be achieved through improved understanding of disease mechanism. Recently, we developed a *Drosophila* model of HPV18E6 plus human E3 ubiquitin ligase (hUBE3A) and demonstrated that the E6-induced cellular abnormalities are conserved between humans and flies. Subsequently, we demonstrated that reduced level and activity of IKKβ, a regulator of NF-κB, suppresses the cellular abnormalities induced by E6 oncoprotein and that the interaction of IKKβ and E6 is conserved in human cells. In this study, we performed transcriptomic analysis to identify differentially expressed genes that play a role in IKKβ-mediated suppression of E6-induced defects. Transcriptome analysis identified 215 genes whose expression was altered due to reduced levels of IKKβ. Of these 215 genes, 151 genes showed annotations. These analyses were followed by functional genetic interaction screen using RNAi, overexpression, and mutant fly strains for identified genes. The screen identified several genes including genes involved in Hippo and Toll pathways as well as junctional complexes whose downregulation or upregulation resulted in alterations of E6-induced defects. Subsequently, RT-PCR analysis was performed for validation of altered gene expression level for a few representative genes. Our results indicate an involvement for Hippo and Toll pathways in IKKβ-mediated suppression of E6 + hUBE3A-induced cellular abnormalities. Therefore, this study enhances our understanding of the mechanisms underlying HPV-induced cancer and can potentially lead to identification of novel drug targets for cancers associated with HPV.

## Introduction

High-risk human papillomaviruses (HR-HPVs) are responsible for almost all cervical cancers and significant number of other HPV-associated cancers including vaginal, vulvar, penile, anal, and oropharyngeal cancers. Annually, more than 750,000 HPV-induced cancer cases are detected from which almost 50% result in death (Pesut *et al.*, 2021). The majority of cases belong to developing countries where the implementation of vaccination and screening strategies have not been fully successful (IARC Working Group on the Evaluation of Carcinogenic Risks to Humans, 2008). The most prevalent types of HR-HPV are HPV16 and 18, which account for the majority of HPV-associated cancers. Most individuals infected with HPV clear the infection within the first 2 years, however, for unknown reasons, this infection persists in a small fraction of the infected population leading to cancer and progression to malignancy. Persistent viral infection results in random integration of viral genome into the host genome. During this event while most viral genes are eliminated, the E6 and E7 viral oncogenes become uncontrollably overexpressed in host's cells, promoting the development of cancerous cells. Although the cooperative action of E6 and E7 is necessary for cellular immortalization and invasive cancer, E7 has been shown to be important in early stages of cancer development while E6 is a key factor in cancer progression and malignancy (Hawley-Nelson *et al.*, 1989; Mantovani and Banks, 2001; Riley *et al.*, 2003). E6 promotes tumor formation and cell immortalization by targeting specific proteins and dysregulating several cellular signaling pathways. These proteins include the tumor suppressor protein p53, the pro-apoptotic protein BAK, and several key regulators of cell polarity and junctions including PDZ domain proteins Dlg, Scribble, and the Magi family of proteins (Scheffner *et al.*, 1990; Scheffner *et al.*, 1993; Gardiol *et al.*, 1999; Glaunsinger *et al.*, 2000; Nakagawa and Huibregtse, 2000; Thomas *et al.*, 2002; Thomas *et al.*, 2005; Kranjec and Banks, 2011). E6 targets these proteins for the proteasomal-mediated degradation with the assistance of a human E3 ubiquitin ligase (hUBE3A) (Huibregtse *et al.*, 1993). Furthermore, E6 dysregulates several signaling pathways, including Wnt, JAK/STAT, Hippo, Notch, and PI3K/AKT/mTOR to promote cell growth, survival, and proliferation (Basukala and Banks, 2021). The exact mechanism of how E6 transforms healthy cells into cancerous ones is not understood in detail, however, the interaction of E6 PDZ binding motif and PDZ domain proteins is crucial for E6-mediated epithelial hyperplasia in vivo (Nguyen *et al.*, 2003).

Lack of detailed understanding of E6-mediated dysregulation of cellular homeostasis is in part related to limitations of in vitro and transgenic mice studies. *In vitro* models do not represent the physiological aspect of the disease and transgenic mice overcome this but are impractical for large-scale genetic and high-throughput compound screening. Hence complementary models that present high conservation of genes and signaling pathways and can overcome these limitations would be highly beneficial. *Drosophila melanogaster* meets these criteria and hence has been serving as an excellent in vivo model system for modeling and studying human diseases including cancer (McGurk *et al.*, 2015; Wangler *et al.*, 2015; Ugur *et al.*, 2016; Sonoshita and Cagan, 2017; Enomoto *et al.*, 2018). Previously, we generated a *Drosophila* model of HPV18E6 plus human UBE3A, which demonstrated conserved mechanisms of E6 actions (Padash Barmchi *et al.*, 2016). Using the advantages of the model such as large-scale in vivo genetic screening, we discovered that reduced levels of IKKβ, a regulator of NF-κB, suppresses the cellular abnormalities induced by E6 oncoprotein and that the interaction of IKKβ and E6 is conserved in human cells (Padash Barmchi *et al.*, 2021). Here, we performed transcriptomic analysis to identify genes whose expression levels are altered due to cooperative action of E6 + hUBE3A and play a role in IKKβ-mediated suppression of E6-induced defects. Our analysis identified 215 genes whose expression were altered due to inhibition of IKKβ. Of these 215 genes, 151 genes showed annotations. Subsequent functional genetic interaction screening using RNAi, overexpression, and mutant fly strains for identified genes identified several genes whose downregulation or upregulation led to alteration of E6-induced defects, suggesting a key role for them in IKKβ-mediated suppression of E6-induced abnormalities. Furthermore, RT-PCR analysis was performed for validation of altered gene expression level for a few representative genes. Our results provide insight into mechanisms by which inhibition of IKKβ blocks the cellular abnormalities induced by the cooperative action of E6 and hUBE3A.

## Materials and methods

### *Drosophila* strains and genetics

The following fly strains have been used: GMR-Gal4, Canton S, and IKKβ^1^/TM6BTb from Bloomington *Drosophila* stock center; UAS-E6, UAS-hUBE3A/Curly Gal80 TM6B Tubby, UAS-E6 (Padash Barmchi *et al.*, 2016), UAS-hUBE3A (Reiter *et al.*, 2006), UAS-trh.WT, P{TRiP.JF01799}attP2 (nmo RNAi), P{TRiP.JF02059}attP2 (vha100-1 RNAi), P{TRiP.JF02267}attP2 (pum RNAi), P{TRiP.HMS01564}attP2 (pum RNAi), UAS-alpha-Cat-GFP, P{TRiP.HMC06634}attP40 (CG10631 RNAi), P{TRiP.HM04026}attP2 (Rtf1 RNAi), P{TRiP.HMS06074}attP2 (Rtf1 RNAi), P{TRiP.GL00015}attP2 (Tao-1 RNAi), P{TRiP.HMS02333}attP40 (Tao-1RNAi), P{TRiP.JF02713}attP2 (MED19 RNAi), P{UAS-Prosβ2^1^}1B, P{TRiP.HM05008}attP2 (mop RNAi), P{TRiP.HMS00706}attP2 (mop RNAi), P{TRiP.JF01421}attP2 (tara RNAi), P{UAS-sqz.A}7-3 were all obtained from Bloomington *Drosophila* stock center.

### RNA isolation and RNA-seq

Total RNA was isolated from pupal eyes 42 h after puparium formation using TRIzol followed by purification by RNeasy Mini kit (QIAGEN). The quality and concentration of RNA were measured using Nanodrop Spectrophotometry. RNA sequencing was performed at the Oklahoma Medical Research Foundation Genomics Center. Prior to RNA-seq analysis quality control measures were implemented. Concentration of RNA was ascertained via fluorometric analysis on a Thermo Fisher Qubit fluorometer. Overall quality of RNA was verified using an Agilent Tapestation instrument. Following initial QC steps mRNA was isolated and libraries were generated using the Swift Biosciences Rapid RNA kit according to the manufacturers protocol. Briefly, mature mRNA was enriched for via pull down with beads coated with oligo-dT homopolymers using the NEBNext Poly(A) mRNA Magnetic Isolation Kit. The mRNA molecules were then chemically fragmented and the first strand of cDNA was generated using random primers incorporating a truncated i5 adapter sequence. Following a bead-based cleanup the 3′ end of the single stranded cDNA was ligated to a truncated i7 adapter. Libraries were then indexed using Swift Biosciences Unique Dual Indexing primers. Final libraries for each sample were assayed on the Agilent Tapestation for appropriate size and quantity. These libraries were then pooled in equimolar amounts as ascertained via fluorometric analyses. Final pools were absolutely quantified using qPCR on a Roche LightCycler 480 instrument with NEB Illumina Library Quantification reagents. Sequencing of the libraries was performed on an Illumina NovaSeq 6000 with PE150 reads. The following genotypes and three replicates of each were subjected to RNA sequencing: (WT), (GMR-Gal4/+), (UAS-E6/GMR-Gal4), (UAS-hUBE3A/GMR-Gal4), (UAS-E6, UAS-hUBE3A/GMR-Gal4), (UAS-E6, UAS-hUBE3A/GMR-Gal4/IKKβ^−^/_+_), and (IKKβ^−^/_+_).

### RNA-seq data analysis

Bioinformatic analyses were performed on the Supercomputing Center for Education & Research (OSCER) at the University of Oklahoma. Adapters were removed using Cutadapt (Martin, 2011), and an initial quality assessment of the raw RNA-seq reads was conducted in FastQC (Andrews, 2010). Given the high quality across all raw reads, no trimming of the data was performed, which is consistent with previous studies suggesting that excessive trimming can have a negative impact on the quality of assemblies and downstream analyses (Macmanes, 2014). RNA-seq reads were aligned to the *Drosophila melanogaster* (Ensembl BDGP5.25) genome using HISAT2 (Kim *et al.*, 2015) with parameters *–dta –max-intronlen 50000*. Transcripts were functionally annotated by blasting the protein sequences against NCBI's Non-Redundant (nr) protein data set, and mapping gene ontology terms using the Blast2GO Basic software (Gotz *et al.*, 2008).

### Differential expression and gene set enrichment analyses

To calculate gene expression, the HISAT2 alignments were used to generate read counts for each gene using the reference genome annotation, the transcript abundances and splice variant identification for all samples was conducted using Cufflinks (Trapnell *et al.*, 2012), and the -N upper-quartile normalization option. Transcripts of genes that were identified by Cufflinks but had no annotation in the *D. melanogaster* genome were searched for open reading frames using TransDecoder (Haas *et al.*, 2013). Differential expression of genes and transcripts was determined using CuffDiff2 (Trapnell *et al.*, 2013) with all three biological replicates, a method that accounts for count overdispersion relative to what would be expected under a Poisson model. Differentially expressed genes were those with a posterior probability >0.95 for a particular expression, which corresponds to an FDR of 0.05. The enrichment analysis was done using tools available in DAVID (Sherman *et al.*, 2022). The conversion of gene ID and statistics of GO term was performed using clusterProfiler (Wu *et al.*, 2021). The circle plot was generated by GOplot (Walter *et al.*, 2015). For GO distribution analysis, the GO terms were mapped using Blast2GO Basic software and GO distribution of differentially expressed genes for biological processes, molecular functions, and cellular components were determined using Fisher's exact test.

### Functional genetic interaction screen

UAS-RNAi, mutant lines, and overexpression lines of genes that were differentially expressed between the (UAS-E6, UAS-hUBE3A/GMR-Gal4) and (UAS-E6, UAS-hUBE3A/GMR-Gal4/IKKβ^−^/_+_) were crossed with (GMR-Gal4, UAS-E6, UAS-hUBE3A/Curly Gal80 TM6B Tubby). The eyes of F_1_ progeny with genotypes containing one copy of (GMR-Gal4, UAS-E6, UAS-hUBE3A) and one copy of the respective UAS-RNAi, mutant, or overexpression gene they were crossed with were compared with the eyes of control flies (GMR-Gal4, UAS-E6, UAS-hUBE3A). In this comparison we searched for functional genetic interaction, which would either result in enhancement or suppression of the E6 + hUBE3A-induced eye abnormalities. Those exhibiting interactions were imaged using an Axioscope. The RNAi and overexpression lines that showed strong functional genetic interactions were then crossed with GMR-Gal4 to determine whether the functional genetic interaction between the genes and E6 + hUBE3A function was independent of the effect of altered expression of those genes alone. The images were compiled and labeled using Adobe Photoshop.

### RT-PCR and gel electrophoresis

cDNAs were synthesized by using and following a QuantiTect cDNA synthesis kit (QIAGEN) and protocol with RNA samples from the following lines: (WT), (GMR-Gal4/+), (GMR-Gal4/UAS-E6, UAS-hUBE3A), and (GMR-Gal4/UAS-E6, UAS-hUBE3A/IKKβ^−^/_+_). The cDNAs were used to follow a QuantiTect reverse transcription polymerase chain reaction kit protocol. Primers for ribosomal protein-49 (RP49) were used as an internal control and primers for genes, which showed functional genetic interaction with E6 + hUBE3A were also used. Gel electrophoresis for the RT-PCRs were performed in a 2.5% agarose gel with SYBR green dye placed in a 1xTAE buffer solution with a 100 base-pair ladder loaded as a reference. Three RT-PCR replicates and gel electrophoresis were performed for each interactive gene. The bands were imaged and the intensity of each band was measured and normalized against the intensity of their corresponding RP49 bands using ImageJ followed by data analysis using prism software to determine if there was a significant change in transcript level between samples.

The following primers obtained from Integrated DNA Technologies were used:

RP49 F: 5′- CCGCTTCAAGGGACAGTATC-3′
RP49 R: 5′- GACAATCTCCTTGCGCTTCT-3′
Tao-1 F: 5′- CATCTCCAATGCAGTCAACG-3′
Tao-1 R: 5′- GCGCACACAGAAATCTACGG-3′
pum F: 5′-GTTGCACGTGATGATGAAGG-3′
pum R: 5′-ATTGATGTGCTTGCCGTAGG-3′
Rtf1 F: 5′-CAATGGTTTGTCCGAGAAGG-3′
Rtf1 R: 5′-CCGTTCTCCTTTGTTCTTGG-3′

## Results

### Alteration in transcriptional profile due to cooperative action of HPV18E6 and human UBE3A

**Fig. 1.**
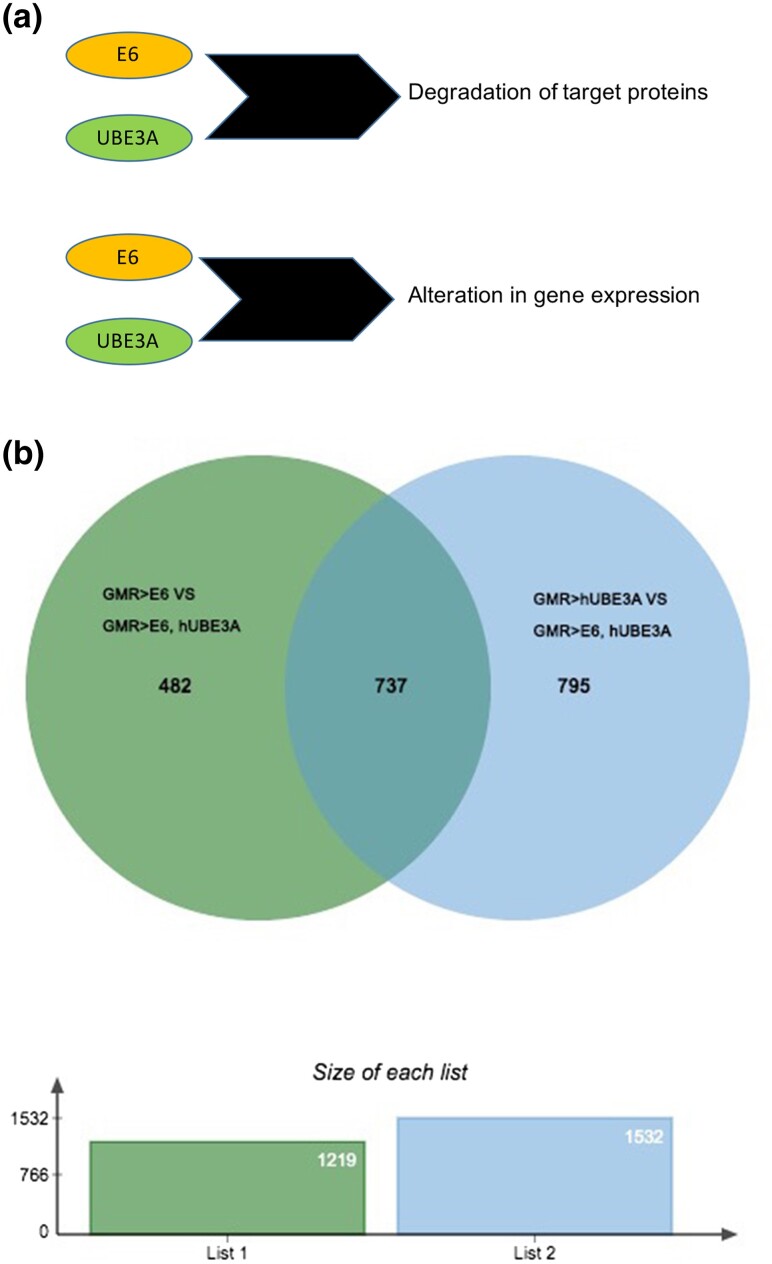
Differentially expressed genes resulting from the combined action of E6 and hUBE3A. a) A model figure showing the cooperative action of E6 and UBE3A in targeting cellular proteins and gene expression. b) The green circle represents 1,219 genes whose expression levels were altered when transcriptome of cells expressing GMR > E6 were compared with transcriptome of cells expressing GMR > E6, hUBE3A. The blue circle displays the 1,532 genes whose expression levels were altered when transcriptome of cells expressing GMR > hUBE3A were compared with transcriptome of cells expressing GMR > E6, hUBE3A. The overlapping area between the green and blue circles denotes 737 genes whose expression levels were altered due to combined effects of E6 + hUBE3A expression.

**Fig. 2.**
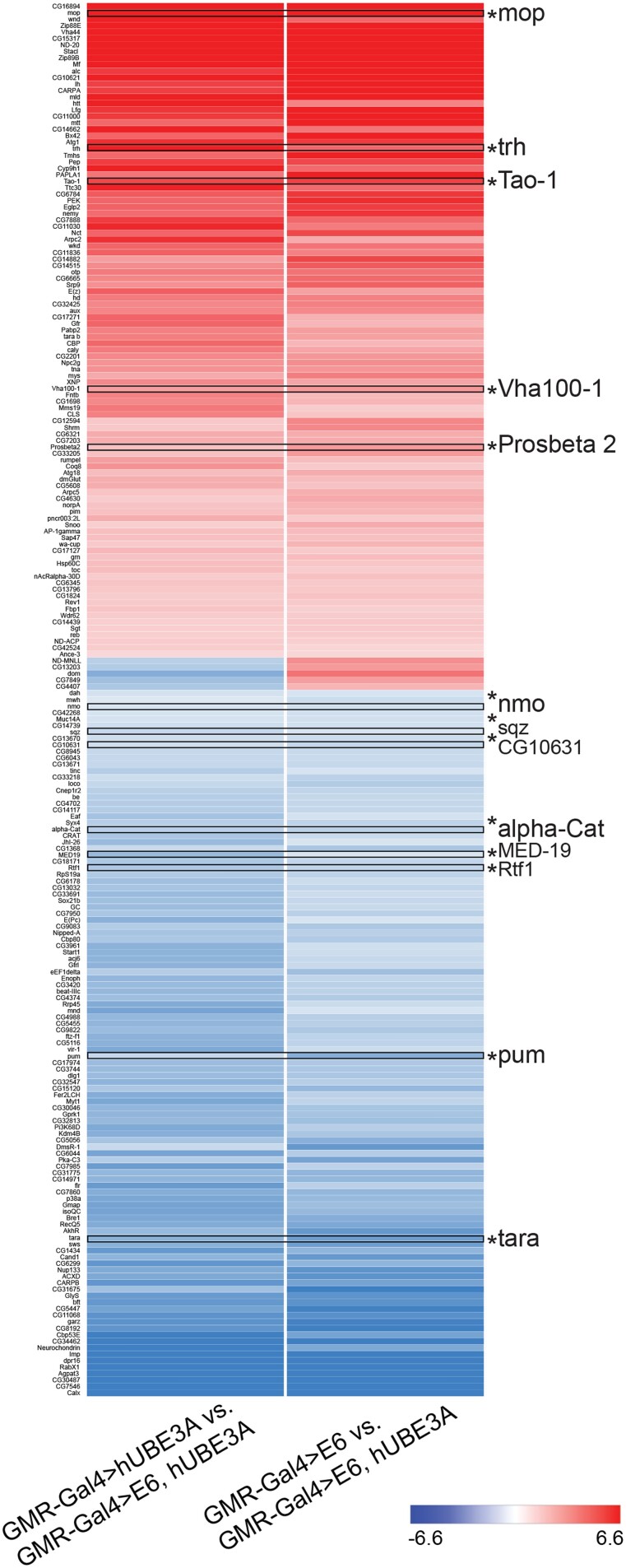
Heat map of the differentially expressed genes between (GMR-Gal4 > hUBE3A vs GMR-Gal4 > E6, hUBE3A) and (GMR-Gal4 > E6 vs GMR-Gal4 > E6, hUBE3A) groups based on fold change values. A negative value corresponds to a downregulated expression shown in blue while a positive value corresponds to an upregulated expression shown in red.

The *Drosophila melanogaster* compound eye consists of 750 eye units known as ommatidia. Ommatidia are epithelial origin and are arranged in stereotype pattern of hexagons. To properly form into functional grids, precise cell proliferation, cell differentiation, and apoptosis are required (Brachmann and Cagan, 2003; Carthew, 2007; Tsachaki and Sprecher, 2012). Disruption of these activities creates a disorganized, rough eye phenotype, which can easily be scored. Previous work has shown that the co-expression of E6 and hUBE3A in developing fly eyes, using an eye-specific Gal4 driver (GMR-Gal4) causes a disorganized, rough eye phenotype accompanied by the degradation of E6 cellular targets and junctional and polarity defects (Padash Barmchi *et al.*, 2016) (Fig. 1a). In order to determine the effect of cooperative action of E6 and hUBE3A on the gene expression, we performed transcriptomic analysis on the pupal eye of flies expressing GMR-Gal4 alone, GMR > UAS-E6 alone, GMR > UAS-hUBE3A alone, and GMR > UAS-E6, UAS-hUBE3A. Three biological replicates were used to control for random fluctuation. The comparison between transcriptome profiling of these genotypes revealed that there were 1,219 genes that were differentially expressed with statistically significant fold change between the GMR > UAS-E6 and GMR > UAS-E6, UAS-hUBE3A (Fig. 1b). Additionally, by comparing the transcriptomes of cells expressing hUBE3A alone with cells expressing E6 + hUBE3A, we found 1,532 genes, which were differentially expressed with statistically significant fold change (Fig. 1b). The results showed that among all these differentially expressed genes, a total of 737 genes presented altered RNA levels that were due to the combined effect of E6 + hUBE3A (the overlapping area between green and blue circles in Fig. 1b). Out of these 737 genes only 337 genes showed annotations (Supplementary Table 1). Differentially expressed genes were those with a posterior probability >0.95 for a particular expression, which corresponds to an FDR of 0.05. Heat map analysis of these differentially expressed genes revealed that 48% of these genes were upregulated while the other 52% were downregulated (Fig. 2). These results were consistent for all three biological replicates suggesting a lack of random fluctuation and a stable gene expression level across replicates. These findings suggest that the cooperative action of E6 and hUBE3A alters the transcript levels of a significant number of genes, either directly or indirectly through targeting cellular proteins that are important for gene expression.

### GO enrichment and distribution analyses of genes that are differentially expressed due to cooperative action of E6 and hUBE3A

**Fig. 3.**
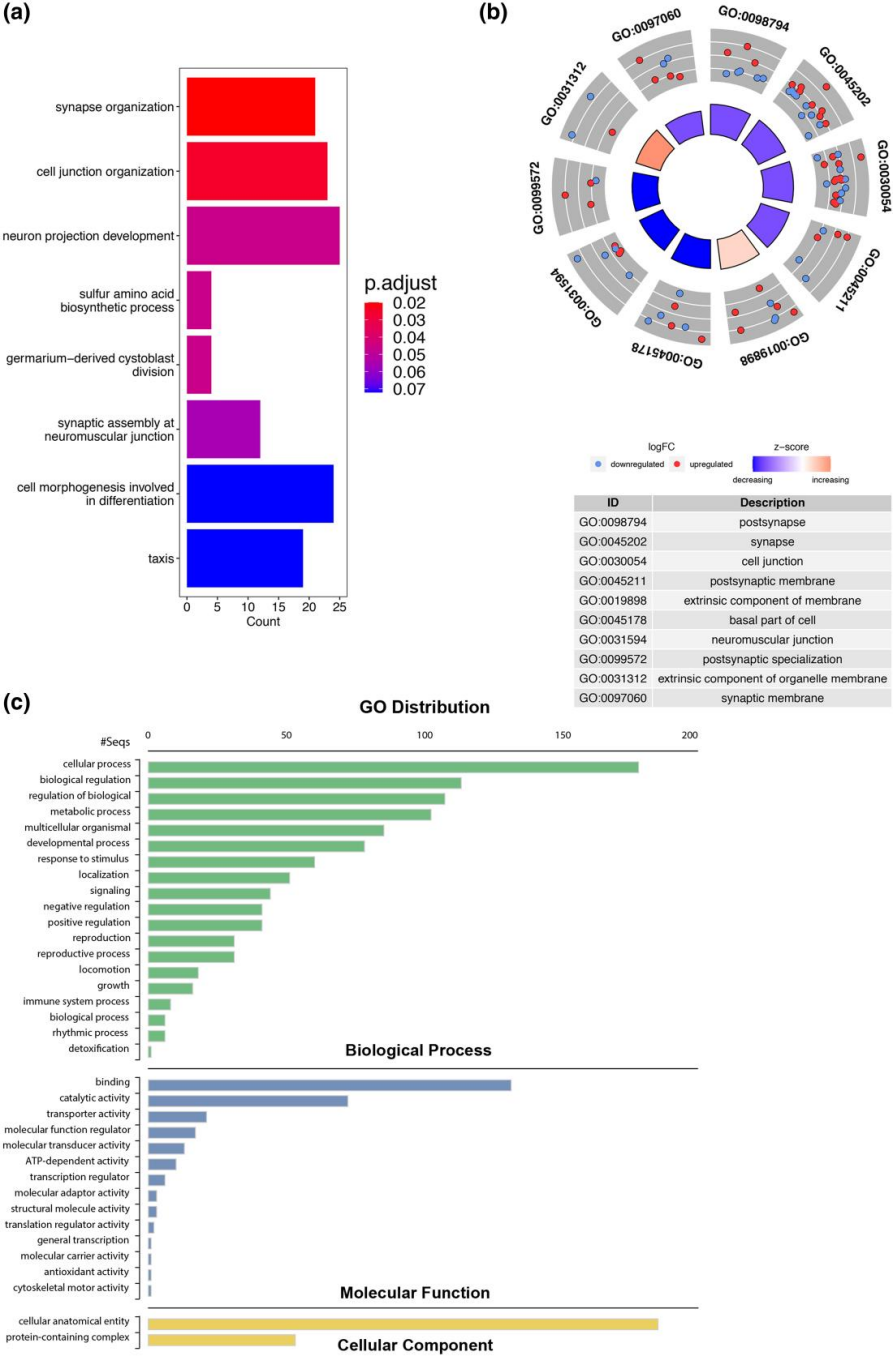
The Gene Ontology (GO) enrichment and distribution analyses of differentially expressed genes due to combined effect of E6 and hUBE3A. a) Biological processes. b) Fold change trend of the top 10 GO terms according to the *P*-value of enrichment. Scatter plots on the outside ring correspond to the fold change (logFC) of genes within one GO term. The inside bar plots correspond to the adjusted *P*-value. The color of the bar is related to the z-score. z-score is the overall fold change of genes. c) The GO distribution. The numbers on the X axis indicates the number of differentially expressed genes belonging to each GO term.

In order to determine to what extent the differentially expressed genes were enriched for biological processes, molecular functions, cellular components, and pathways we performed GO enrichment analysis using tools available from DAVID (Huang *et al.*, 2009). Our analyses revealed that 5 GO terms among biological processes including synapse organization, cell junction organization, neuron projection development, sulfur amino acid biosynthetic process, and germarium-derived cystoblast division were significantly enriched (Fig. 3a and Supplementary Table 3). For cellular components, molecular functions, and KEGG pathway we did not detect any significant enrichment. However, GO terms including postsynapse, synapse, and cell junction were over-represented for cellular components according to p-value (Supplementary Fig. 1 and Supplementary Table 3). We also found that each of these GO categories possessed a combination of downregulated and upregulated genes (Fig. 3b). We next conducted a gene ontology distribution analysis to determine which cellular functions were most altered due to co-expression of E6 and hUBE3A. Our result showed that the differentially expressed genes were involved in a range of cellular functions and processes among which the “cellular process” category, which includes many cellular activities such as cell cycle, cell communication, cell adhesion, and cell death was the most represented followed by “biological regulation”, “regulation of biological process”, and “metabolic process” (Fig. 3c). The majority of the differentially expressed genes exhibited binding and catalytic activity (Fig. 3c) suggesting that alteration in expression level and activity of proteins that act as catalysts is an important function of E6 and hUBE3A in navigating the cell toward cancerous status.

### Reduced level of IKKβ alters the transcriptional profile induced by cooperative action of E6 and hUBE3A

**Fig. 4.**
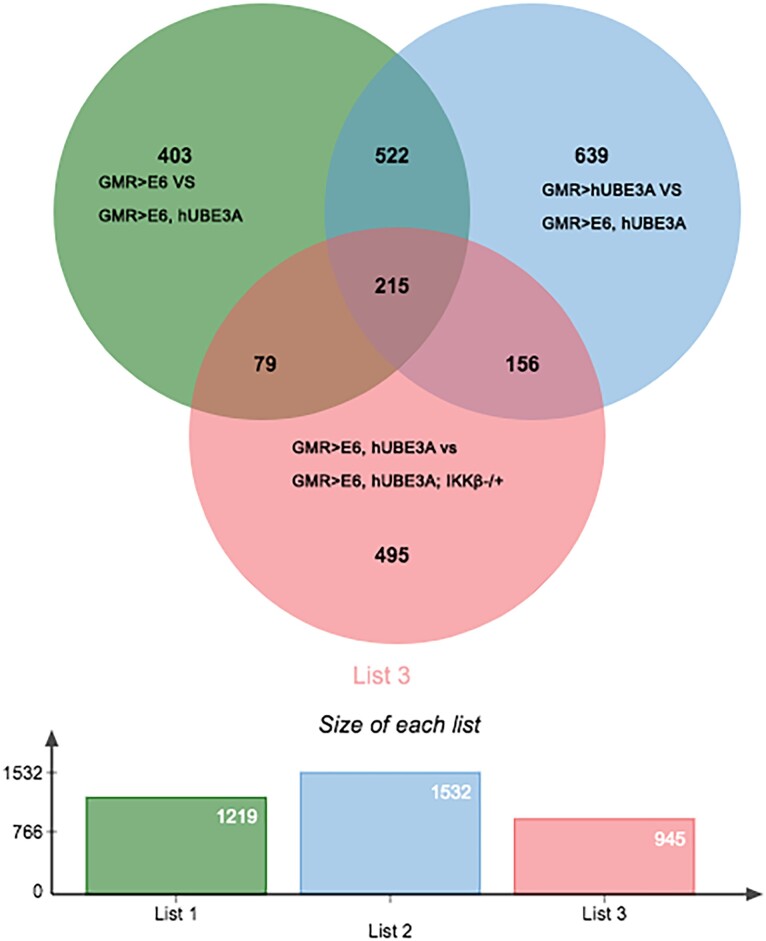
Differentially expressed genes due to reduced level of IKKβ in the presence of E6 and hUBE3A. The green circle represents 1,219 genes whose expression levels were altered when transcriptome of cells expressing GMR > E6 were compared with transcriptome of cells expressing GMR > E6, hUBE3A. The blue circle displays the 1,532 genes whose expression levels were altered when transcriptome of cells expressing GMR > hUBE3A were compared with transcriptome of cells expressing GMR > E6, hUBE3A. The red circle indicates the 945 genes with altered expression between GMR > E6, hUBE3A and GMR > E6, hUBE3A; IKKβ-/+. 215 genes overlapped by all three circles represents those, which were altered due to reduced level of IKKβ within E6 + hUBE3A-expressing cells.

**Fig. 5.**
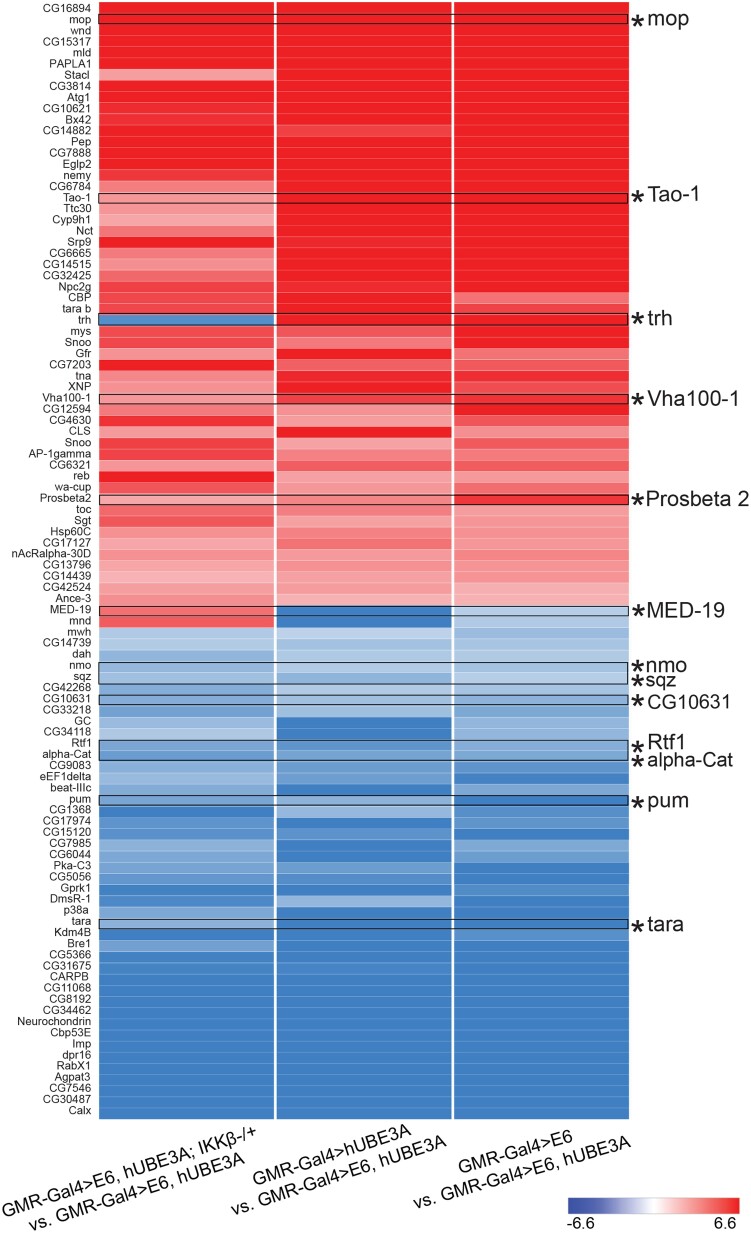
Heat map of the differentially expressed genes between (GMR-Gal4 > E6, hUBE3A; IKKβ-/+ vs GMR-Gal4 > E6, hUBE3A), (GMR-Gal4 > hUBE3A vs GMR-Gal4 > E6, hUBE3A), and (GMR-Gal4 > E6 vs GMR-Gal4 > E6, hUBE3A) groups based on fold change values. A negative value corresponds to a downregulated expression shown in blue while a positive value corresponds to an upregulated expression shown in red.

Our recent study has shown that the reduced expression of IKKβ leads to suppression of the cellular abnormalities induced by cooperative action of E6 and hUBE3A and that the interaction of IKKβ and E6 is conserved in human cells (Padash Barmchi *et al.*, 2021). In order to identify the genes that play a role in IKKβ-mediated suppression of E6-induced defects, we compared the transcriptome profile of the cells expressing E6 + hUBE3A and cells expressing E6 + hUBE3A with a reduced level of IKKβ. We identified 945 genes whose transcript levels were altered (Fig. 4) with a fold change that was statistically significant (FDR of 0.05). Among these 945 genes the transcript levels of 215 genes that were altered due to cooperative action of E6 and hUBE3A were affected in the presence of reduced level of IKKβ (Fig. 4, the overlapping area between green, blue, and red circles). Of these 215 genes, 151 genes showed annotations (Supplementary Table 2). Heat map analysis of the most conserved differentially expressed genes revealed that 97% of these genes showed a reverse expression level change, with 53% of them upregulated and 44% downregulated, when compared with their expression levels in the cells expressing E6 + hUBE3A. Furthermore, we found that the transcript levels of 2% of differentially expressed genes were further increased whilst 1% were further decreased in the presence of reduced levels of IKKβ in cells co-expressing E6 and hUBE3A (Fig. 5). These results were consistent for all three biological replicates suggesting a lack of random fluctuation and a stable gene expression level across replicates.

### GO enrichment and distribution analyses of genes that are differentially expressed when the level of IKKβ is reduced in the E6 + hUBE3A-expressing cells

**Fig. 6.**
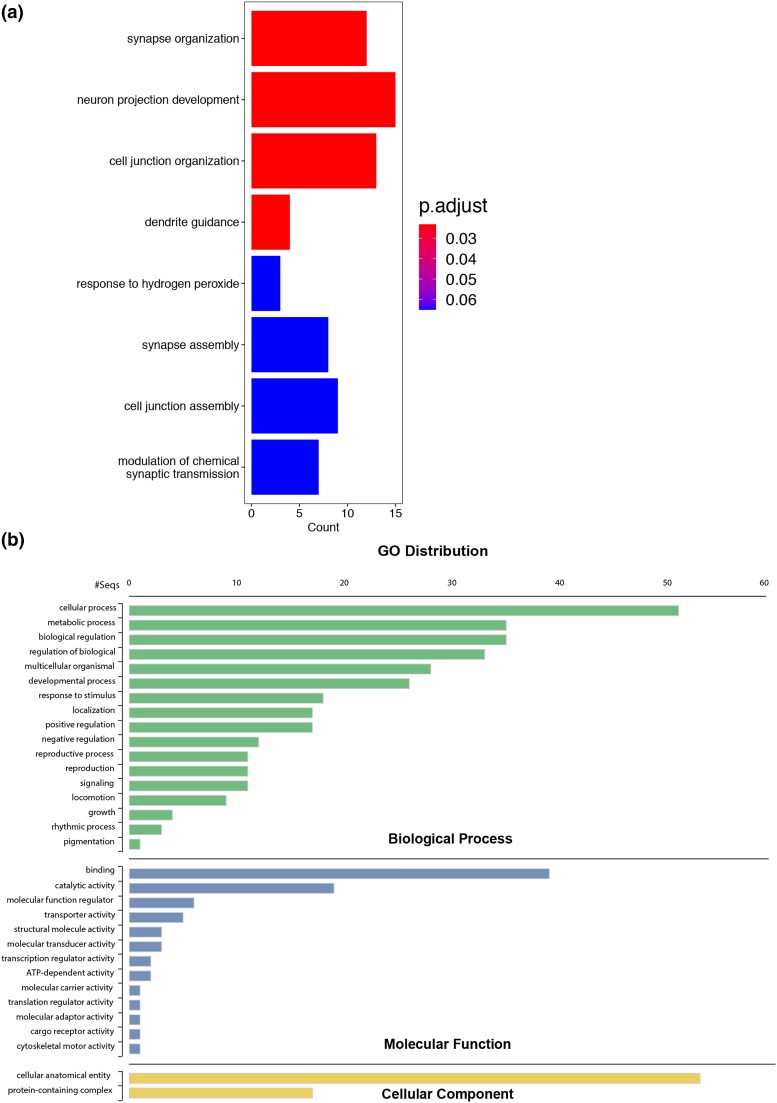
The Gene Ontology (GO) enrichment and distribution analyses of differentially expressed genes when the level of IKKβ is reduced in the E6 + hUBE3A-expressing cells. a) Biological processes. b) GO distribution. The numbers on the X axis indicates the number of differentially expressed genes belonging to each GO term.

In order to determine which biological functions were over-represented within the differentially expressed gene set we conducted ontological enrichment analysis using DAVID algorithm. Our analysis showed that biological processes including synapse organization, neuron projection development, cell junction organization, and dendrite guidance were the most prominent ontologies that were significantly enriched (Fig. 6a and Supplementary Table 4). GO enrichment analysis for cellular components, molecular functions, and KEGG pathway did not result in any significant enrichments (Supplementary Fig. 2 and Supplementary Table 4). Furthermore, GO distribution analysis revealed that most of the genes with altered expression due to the interaction between E6 + hUBE3A and reduced IKKβ levels were involved in cellular process, metabolic process, regulation of biological process, and biological regulation (Fig. 6b). Additionally, genes whose proteins possess binding, catalytic, and transporter activities were most represented (Fig. 6b). This finding was also noted in GO enrichment analysis for molecular functions as GO terms including metal ion binding, actin filament binding and GO terms belonging to enzyme category, were among the top ontologies (Supplementary Fig. 2b). These results suggest that reduced level and activity of IKKβ interferes with E6 + hUBE3A action in altering several cellular functions within ommatidial units including those involved in photoreceptor development and junctional organization. Furthermore, genes whose products act as enzymes including kinases and those with transporter activities are likely to be key players in IKKβ-mediated suppression of E6 + hUBE3A-induced cellular abnormalities.

### Functional genetic screen identifies several of the differentially expressed genes as important players in the IKKβ-mediated suppression of E6-induced defects

**Fig. 7.**
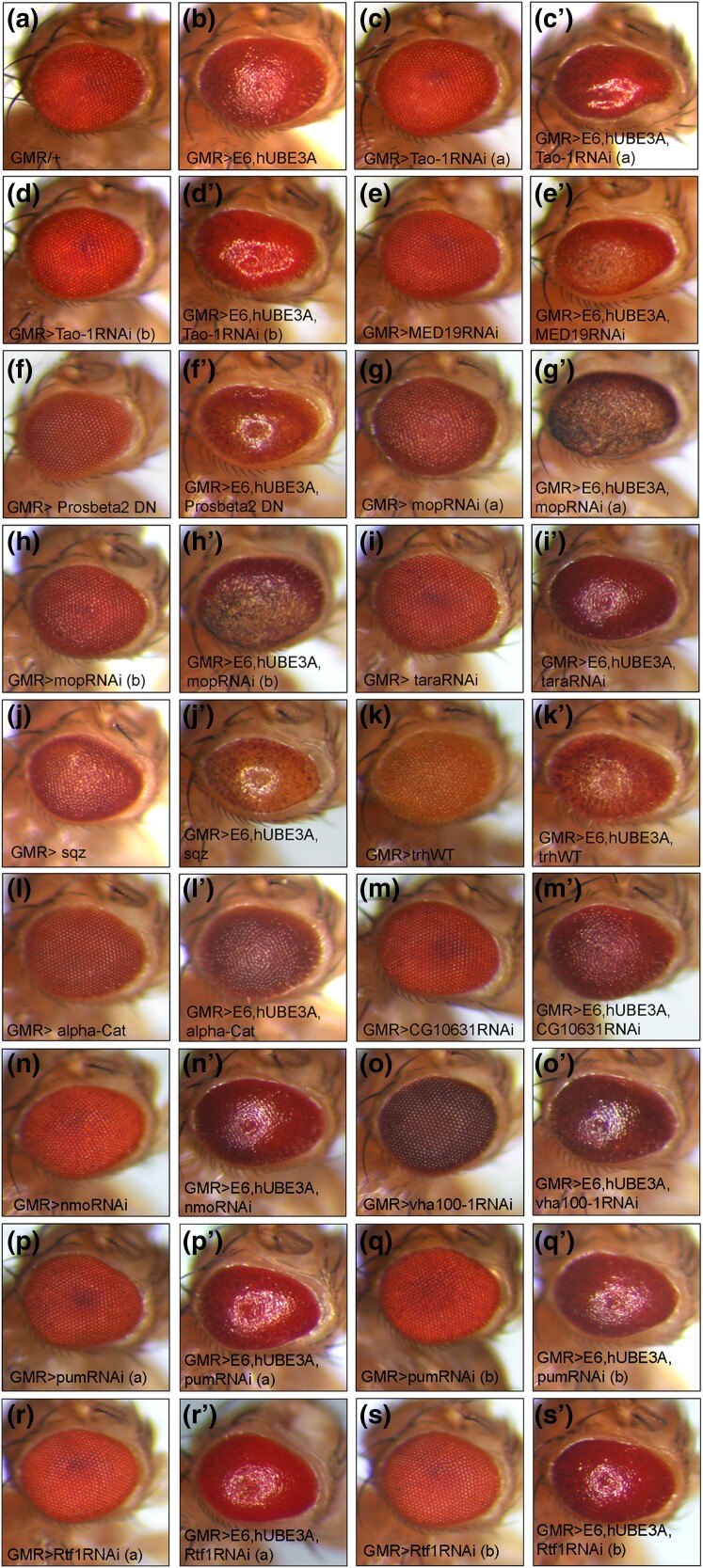
IKKβ-mediated differentially expressed genes display functional genetic interaction with E6 + hUBE3A. GMR-Gal4/+ showing normal eye phenotype (a). Fly eye expressing E6 and hUBE3A driven by GMR-Gal4. The eye exhibits rough morphology (b). These two lines were used as control strains for functional genetic screen to identify the enhancers and suppressors of E6 + hUBE3A-induced morphological abnormalities. From the screen, increased expression of alpha-Cat and downregulation of CG10631 using RNAi resulted in partial suppression of E6 + hUBE3A-induced eye defects (l’ and m’ compared with b). The expression of alpha-Cat or downregulation of CG10631 alone in the absence of E6 + hUBE3A expression did not result in any eye morphological changes (l and m). Downregulation of MED19 (e’), expression of a dominant negative form of Prosbeta 2 (f’), downregulation of mop (g’ and h’), tara (i’), increased expression of sqz (j’) and trh (k’), downregulation of nmo (n’), vha100-1 (o’), pum (*p*’ and q’), and Rtf1 (r’ and s’) all resulted in enhancement of E6 + hUBE3A-induced eye morphological defects. Control eyes shown in (c, d, e, f, g, h, i, j, k, n, o, p, q, r, s) show that the interaction of differentially expressed genes with E6 + hUBE3A were specific as alterations in the level of these genes alone in the absence of E6 + hUBE3A did not result in those phenotypes. (a) and (b) indicate independent RNAi strains.

In order to determine which of the 151 annotated genes whose expression levels were altered due to reduced level of IKKβ were important players in IKKβ-mediated suppression of E6-induced defects, we performed a functional genetic interaction screen using available RNAi, mutant, and overexpression strains against all differentially expressed genes with homology to human counterpart. In this screen we crossed the GMR-Gal4, UAS-E6, UAS-hUBE3A/Curly Gal80 TM6B Tubby virgin females with males of RNAi, mutant, and overexpression strains of the differentially expressed genes. The eyes of F_1_ progeny with genotypes containing one copy of GMR-Gal4, UAS-E6, UAS-hUBE3A and a copy of the respective UAS-RNAi, mutant, or overexpression gene were compared with the eyes of control flies expressing GMR-Gal4, UAS-E6, UAS-hUBE3A. In this comparison, we searched for functional genetic interaction, which would either result in enhancement or suppression of the E6 + hUBE3A-induced eye abnormalities. Through this screen we were able to identify 13 genes, which showed strong functional interactions with E6 + hUBE3A (Fig. 7). The remaining tested genes did not show any functional interactions. Of 13 identified genes, 8 were downregulated and 5 were upregulated when eye tissues expressing either E6 or hUBE3A were compared with eye tissues expressing E6 + hUBE3A (highlighted genes in Fig. 2). Similarly comparing RNA profile of eye tissues expressing E6 + hUBE3A + one functional genomic copy of IKKβ with RNA profile of eye tissues expressing E6 + hUBE3A revealed that of 13 genes 5 showed increased expression whilst the remaining 8 genes were downregulated (highlighted genes in Fig. 5). Of the identified interactors, overexpression of alpha-Catenin (a regulator of adherens junction), and RNAi-mediated knockdown of CG10631 showed phenotypic rescues whilst the other 11 genes when downregulated or upregulated enhanced E6 + hUBE3A-induced eye defects (Fig. 7). Notably, among enhancers, myopic (mop) knock down in the E6 + hUBE3A-expressing eye tissue, using several independent RNAi lines, resulted in transformed eye morphology with the presence of necrotic tissue (Fig. 7g’ and h’ compared with b). Similarly, downregulation of Tao-1, a Ser/Thr kinase, using multiple independent RNAi lines resulted in formation of tissue folding (Fig. 7c’ and d’ compared with b). Both of these genes are negative regulators of the transcriptional co-activator Yorkie/YAP1 (Yes-associated protein 1) within Hippo pathway whose activity is important for expression of genes involved in cell proliferation, growth, and survival (Boggiano *et al.*, 2011; Poon *et al.*, 2011; Yu *et al.*, 2015; Verghese and Moberg, 2019). Furthermore, we found that the functional genetic interaction between these 13 genes and E6 + hUBE3A were specific as alterations in the level of these genes alone when E6 + hUBE3A expression was absent did not result in those phenotypes (Fig. 7).

**Fig. 8.**
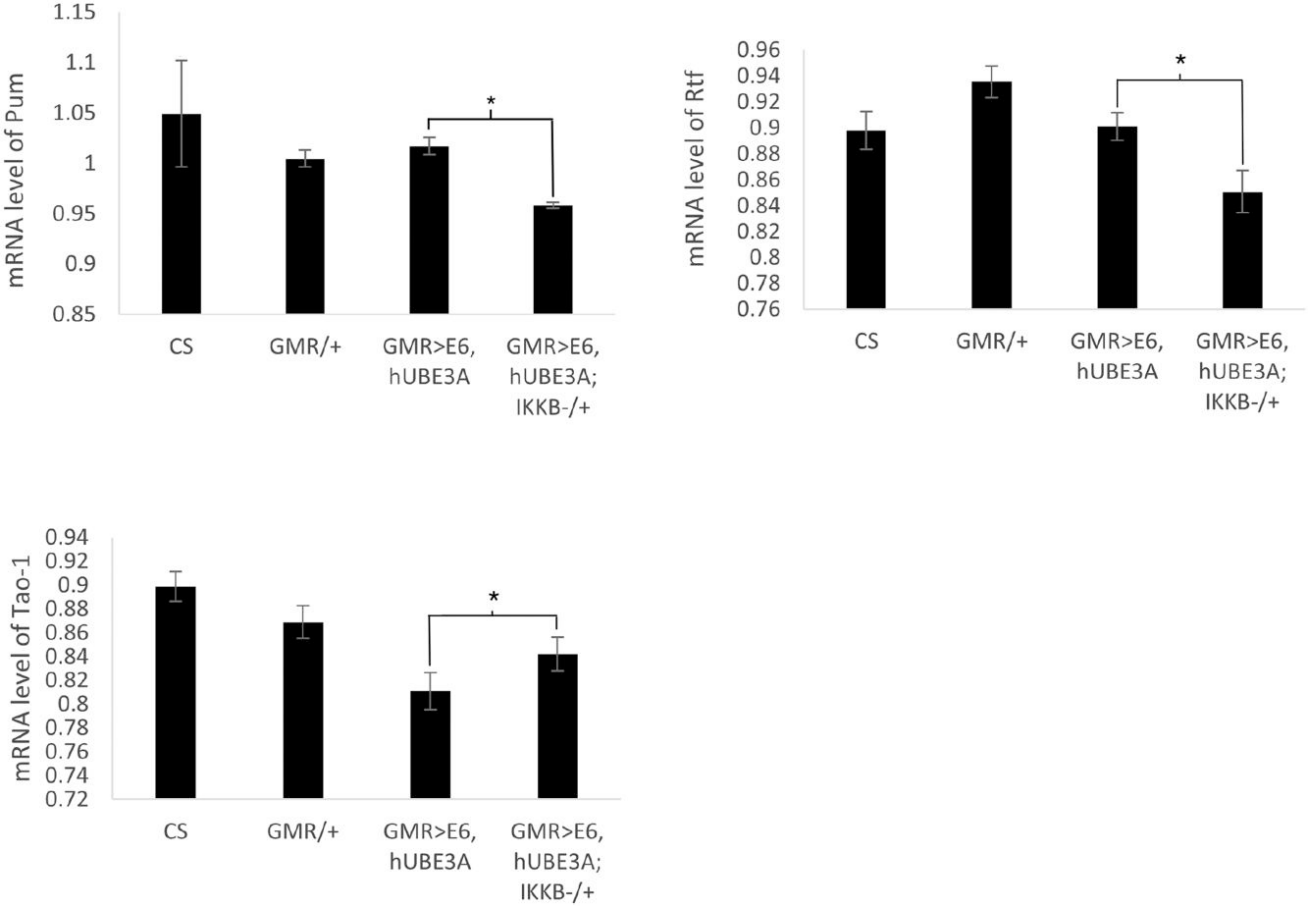
Validation of altered expression level of differentially expressed genes using RT-PCR. The intensity of each band was measured and normalized against its corresponding RP49 band intensity. Error bars indicate the SEM of 3 biological replicates and asterisk (*) denotes the statistically significant difference (*P* < 0.05).

Next we sought to validate the altered expression level of several differentially expressed genes by performing RT-PCR. These analyses were performed for a few represented genes including Tao-1, Rtf1, and pum. Consistent with our RNA-Seq results we found that while the transcript level of Tao-1 was increased, the RNA level of Rtf1 and pum were decreased in cells expressing E6 + hUBE3A with a reduced level of IKKβ (Fig. 8).

Altogether, these results indicate that reduction in the level of IKKβ suppresses tissue and cellular abnormalities associated with cooperative action of E6 + UBE3A through regulation of several signaling pathways including Hippo as well as cell junctions, which itself is a regulator of Hippo signaling (Silvis *et al.*, 2011; Karaman and Halder, 2018; Sarpal *et al.*, 2019).

## Discussion

In this study, we identified 215 genes whose E6 + hUBE3A-mediated altered expression levels were affected when IKKβ was reduced suggesting a role for them in IKKβ-mediated suppression of E6 + hUBE3A-induced cellular abnormalities. Of these 215 genes, 151 genes indicated annotations, which were considered for analyses. The functional genetic screen analysis revealed that 13 of these genes when upregulated or downregulated, resulted in suppression or enhancement of E6 + hUBE3A-induced eye defects. Out of 13 identified genes, alpha-Catenin overexpression and CG10631RNAi exhibited partial suppression of E6 + hUBE3A-induced morphological defects. Given that alpha-Catenin expression was downregulated in tissues expressing E6 + hUBE3A + one functional genomic copy of IKKβ, this finding was surprising. However, previous studies have shown that specific functions of alpha-Catenin is dose-dependent (Sarpal *et al.*, 2019). Alpha-Catenin is a cell-cell junction protein found within catenin-cadherin complex that promote cell adhesion. Alpha-Catenin interacts with E-cadherin as well as β-catenin to connect the cadherin-catenin complex to actin cytoskeleton and stabilize the cell adhesion (Nelson, 2008). In addition to its role in cell adhesion, alpha-Catenin possess mechanosensing activities, which can lead to reorganization of actin cytoskeleton and modulation of tissue growth through regulation of Hippo pathway (Rauskolb *et al.*, 2014; Ishiyama *et al.*, 2018). Changes in mechanical tension will promote alpha-Catenin to sequester Ajuba (Jub)-Wts complex at cell junction, which in turn results in Yorkie activation and tissue growth (Silvis *et al.*, 2011; Alegot *et al.*, 2019). This function of alpha-Catenin is dependent on the amount of alpha-Catenin at cell junction. Moderate overexpression or reduction of alpha-Catenin result in tissue overgrowth whilst strong reduction leads to tissue undergrowth (Sarpal *et al.*, 2019). Hence, the presence of higher levels of alpha-Catenin in E6 + hUBE3A-expressing cells is likely due to E6 action in promoting tissue growth and cell survival through activation of Yorkie. Consistent with this view we found a significant reduction in the level of alpha-Catenin in tissues expressing E6 + hUBE3A + one functional genomic copy of IKKβ. This strong downregulation of alpha-Catenin is expected as it likely leads to inactivation of Yorkie and normalization of tissue growth and size, a phenotype that was observed when IKKβ was reduced in eye tissues expressing E6 + hUBE3A (Padash Barmchi *et al.*, 2021). alpha-Catenin has also been shown to act as tumor suppressor in breast cancer cells by interacting with IκBα, which in turn results in stabilization of IκBα and inhibition of the NF-κB signaling (Piao *et al.*, 2014). Imbalance of β-catenin has been reported to contribute to cervical cancer (Perez-Plasencia *et al.*, 2008) and IKKβ phosphorylation of β-catenin leads to its ubiquitination and subsequent proteasomal-mediated degradation resulting in impaired Wnt signaling and cell adhesive properties (Lamberti *et al.*, 2001). Hence, E6 + hUBE3A-induced alterations in the level of alpha-Catenin and the effect of reduced IKKβ level on this alteration is expected in our study. Reduced IKKβ could result in stabilization of catenin-cadherin complex leading to a suppression of cell adhesion and Wnt signaling defects. Altogether, alpha-Catenin presents an interesting candidate to be explored further in order to elucidate the mechanism of IKKβ-mediated suppression of E6 + hUBE3A-induced cellular abnormalities. The CG10631 gene, which is the *Drosophila* homolog of human zinc finger protein 212 and zinc finger protein 671 is predicted to act as transcription factor that negatively regulates gene expression.

Another differentially expressed gene whose E6 + hUBE3A-induced altered expression was affected in the presence of reduced level of IKKβ was myopic (mop). Our functional genetic interaction studies revealed that knockdown of mop using multiple independent RNAi transgenic strains led to an enhancement of E6 + hUBE3A-induced morphological defects. mop is the *Drosophila* homolog of the human PTPN23 and plays a role as a member of the protein tyrosine phosphatase family (Toyooka *et al.*, 2000). The region of the chromosome that contains PTPN23 gene is frequently deleted in cancers (Kok *et al.*, 1997; Braga *et al.*, 2002). The endocytic function of mop has been shown to play important role in several signaling pathways involved in growth, proliferation, and apoptosis including EGFR, Toll and Hippo (Ferrandon *et al.*, 2007; Miura *et al.*, 2008; Gilbert *et al.*, 2011). Mop physically interacts with Yorkie, and restricts its activity (Gilbert *et al.*, 2011). Found within the Hippo signaling pathway, Yorkie is a co-activator of cell growth and has anti-apoptosis properties when found within the nucleus, but not when retained in the cytosol. Hence an increase in mop level in cells expressing E6 + hUBE3 plus a reduced level of IKKβ is likely involved in restricting the activity of Yorkie. This function will result in limiting the cell proliferation as loss of mop has been shown to cause an increased cell division (Gilbert *et al.*, 2011). This is consistent with overgrowth phenotype of RNAi knock down of mop in eye tissue expressing E6 + hUBE3A (Fig. 7g’ and h’). Additionally, given the role of mop in regulation of Toll and immune deficiency (IMD) pathways as well as IKKβ phosphorylation of Relish (Silverman *et al.*, 2000), the *Drosophila* NF-κB transcription factor in IMD pathway, it is plausible that alteration in the level of mop is due to effect of reduced IKKβ on innate immune pathways.

Tao-1 was another differentially expressed gene whose E6 + hUBE3A-induced altered expression was affected in the presence of reduced level of IKKβ. We found that downregulation of Tao-1 using multiple independent RNAi strains resulted in formation of tissue folds in eyes co-expressing E6 and hUBE3A. Tao-1 is the *Drosophila* homolog of the human TAOK, a Ser/Thr kinase belonging to Mst/Ste20 family. Similar to Mop, Tao-1 has been shown to restrict tissue growth through phosphorylation of Hippo, which results in its activation and subsequent inhibition and cytoplasmic retention of Yorkie (Poon *et al.*, 2011). Therefore, the tissue overgrowth phenotype of the E6 + hUBE3A-expressing eye tissue when mop or Tao-1 are downregulated is in part due to activation of Yorkie, its nuclear localization and activation of growth-inducing genes. Consistent with these results, an increase in Tao-1, similar to mop is likely to lead to a restricted growth in tissue expressing E6 + hUBE3A plus a reduced level of IKKβ. Since both Tao-1 and Mop are important regulators of the Hippo signaling pathway, they are excellent candidates for further research to understand their role in IKKβ-mediated suppression of E6 + hUBE3A-induced cellular abnormalities.

In summary, our study has identified several genes some of which operate in the same pathways to enhance or suppress E6 + hUBE3A-induced cellular defects. Given their high conservation and implication in human cancer, these genes are excellent candidates for further investigations in understanding the mechanism of HPVE6 + hUBE3A-induced cellular abnormalities.

## Supplementary Material

jkad020_Supplementary_Data

## Data Availability

Raw RNA-seq reads are available through NCBI BioProject accession number PRJNA879794. The results of the analyses are available in excel file format as Supplementary material. Supplemental material available at G3 online.
