# Supplementary material for: Transcriptomic analysis provides insight into the mechanism of IKKβ-mediated suppression of HPV18E6-induced cellular abnormalities
Source: G3 (Bethesda). 2023 Feb 1;13(4):jkad020. doi: 10.1093/g3journal/jkad020 (PMC10085804; doi:10.1093/g3journal/jkad020)
Supplement: jkad020_Supplementary_Data [file jkad020_supplementary_data.zip › Supplementary_figures-legends_G3-2022-404034.docx]

**Supplementary Figure Legends**

**Figure S1.** The Gene Ontology (GO) enrichment analysis of differentially expressed genes due to combined effect of E6 and hUBE3A. (A) Cellular components. (B) Molecular functions. (C) KEGG pathway. All figures represent top GO terms based on smallest adjusted p-value of enrichment. The numbers on the X axis indicates the number of differentially expressed genes belonging to each GO term.

**Figure S2.** The Gene Ontology (GO) enrichment analysis of differentially expressed genes when the level of IKKβ is reduced in the E6+hUBE3A-expressing cells. (A) Cellular components. (B) Molecular functions. (C) KEGG pathway. All figures represent top GO terms based on smallest adjusted p-value of enrichment. The numbers on the X axis indicates the number of differentially expressed genes belonging to each GO term.
